# Altered Global Synchronizations in Patients With Parkinson’s Disease: A Resting-State fMRI Study

**DOI:** 10.3389/fnagi.2019.00139

**Published:** 2019-06-25

**Authors:** Mengyan Li, Yanjun Liu, Haobo Chen, Guihe Hu, Shaode Yu, Xiuhang Ruan, Zhenhang Luo, Xinhua Wei, Yaoqin Xie

**Affiliations:** ^1^Department of Neurology, Guangzhou First People’s Hospital, School of Medicine, South China University of Technology, Guangzhou, China; ^2^Institute of Biomedical and Health Engineering, Shenzhen Institutes of Advanced Technology, Chinese Academy of Sciences, Shenzhen, China; ^3^Department of Radiation Oncology, Southwestern Medical Center, University of Texas, Dallas, TX, United States; ^4^Department of Radiology, Guangzhou First People’s Hospital, School of Medicine, South China University of Technology, Guangzhou, China; ^5^GYENNO Technologies Co., Ltd., Shenzhen, China

**Keywords:** resting-state fMRI, global synchronizations, Parkinson’s disease, cognitive function, movement function

## Abstract

**Background**: Abnormalities of cognitive and movement functions are widely reported in Parkinson’s disease (PD). The mechanisms therein are complicated and assumed to a coordination of various brain regions. This study explored the alterations of global synchronizations of brain activities and investigated the neural correlations of cognitive and movement function in PD patients.

**Methods**: Thirty-five age-matched patients with PD and 35 normal controls (NC) were enrolled in resting-state functional magnetic resonance imaging (rs-fMRI) scanning. Degree centrality (DC) was calculated to measure the global synchronizations of brain activity for two groups. Neural correlations between DC and cognitive function Frontal Assessment Battery (FAB), as well as movement function Unified Parkinson’s Disease Rating Scale (UPDRS-III), were examined across the whole brain within Anatomical Automatic Labeling (AAL) templates.

**Results**: In the PD group, increased DC was observed in left fusiform gyrus extending to inferior temporal gyrus, left middle temporal gyrus (MTG) and angular gyrus, while it was decreased in right inferior opercular-frontal gyrus extending to superior temporal gyrus (STG). The DC in a significant region of the fusiform gyrus was positively correlated with UPDRS-III scores in PD (*r* = 0.41, *p* = 0.0145). Higher FAB scores were shown in NC than PD (*p* < 0.0001). Correlative analysis of PD between DC and FAB showed negative results (*p* < 0.05) in frontal cortex, whereas positive in insula and cerebellum. As for the correlations between DC and UPDRS-III, negative correlation (*p* < 0.05) was observed in bilateral inferior parietal lobule (IPL) and right cerebellum, whereas positive correlation (*p* < 0.05) in bilateral hippocampus and para-hippocampus gyrus (*p* < 0.01).

**Conclusion**: The altered global synchronizations revealed altered cognitive and movement functions in PD. The findings suggested that the global functional connectivity in fusiform gyrus, cerebellum and hippocampus gyrus are critical regions in the identification of cognitive and movement functions in PD. This study provides new insights on the interactions among global coordination of brain activity, cognitive and movement functions in PD.

## Introduction

As one of the most common neurodegenerative diseases, people diagnosed with Parkinson’s disease (PD) are widely reported with abnormalities consisting of motor and non-motor symptoms (Chaudhuri et al., 2006; Bunzeck et al., 2013; Villarreal et al., 2018). PD is known as a kind of movement disorder, including general motor symptoms and specific motor symptoms (Fox et al., 2018). However, non-movement aspects such as impairment of cognitive and executive functions have also gained great attention and have been the topic of a great number of researches on PD (Kudlicka et al., 2011; Litvan et al., 2011; Dirnberger and Jahanshahi, 2013; Delgado-Alvarado et al., 2016). Various methods are performed to explore the biomarkers for the diagnosis and progression monitoring of PD, including metabolomics profiling of blood (Bogdanov et al., 2008), cerebrospinal fluid (Hong et al., 2010), cognitive impairment (Svenningsson et al., 2012) and neuroimaging (Reijnders et al., 2010; Tessitore et al., 2016; Li et al., 2018).

Structural and functional changes in patients with PD are observed in many neuroimaging studies (Borroni et al., 2015; Wang et al., 2016; Prell, 2018). Structural changes are reported in various brain regions such as corpus callosum, hippocampus, basal ganglia, temporal cortex and frontal cortex by voxel-based morphometry (VBM) analysis (Camicioli et al., 2003; Summerfield et al., 2005; Wiltshire et al., 2010; Tessitore et al., 2016; Lee et al., 2018; Prell, 2018). In recent years, resting-state functional magnetic resonance imaging (rs-fMRI) has become a prevalent method to explore the alterations of spontaneous brain activities (Fox and Raichle, 2007; Van Eimeren et al., 2009) in patients with PD. For the functional neuroimaging aspect, the blood oxygen level dependent (BOLD) signal is widely employed to explore the differences of spontaneous brain activity between healthy people and PD patients (Göttlich et al., 2013; Pan et al., 2017a; Wang et al., 2018). Functional neuroimaging studies of PD are mainly focused on the spontaneous brain activity of amplitude of low frequency oscillations (Kwak et al., 2012; Hou et al., 2014; Pan et al., 2017b), regional synchronization (Wu et al., 2009; Li et al., 2016; Pan et al., 2017a) and functional connectivity (de Schipper et al., 2018; Wang et al., 2018).

The neuroimaging findings of PD are various and inconsistent. In the current stage, it is still unclear which structural or functional neuroimaging marker is reliable or convincing for understanding the pathological physiology of PD. While these findings suggest that the pathophysiological mechanisms in PD is complicated and assumed to a coordination of various brain regions. Degree centrality (DC), a voxel-wise measurement, is applied to evaluate the strengths of functional connectivity across the whole brain (Buckner et al., 2009). DC is a reliable rs-fMRI indicator (Zuo et al., 2013) and suggested to represent the global synchronizations or global functional connectivity density (Tomasi and Volkow, 2012). Using the DC method, alterations are found in brain regions associated with cognition and motor, with these regions being depressed in PD sufferers (Wang et al., 2018). Higher degrees are observed in the precuneus in PD patients with cognitive impairments than patients without cognitive impairment (Nagano-Saito et al., 2019). In the current study, we applied DC to investigate the differences of global synchronizations of brain activity between PD and normal controls (NC). Furthermore, the neural correlations between global synchronizations and cognitive function as well as movement function were explored in PD group across the whole brain.

## Materials and Methods

### Participants

In this study, 35 NC and 35 patients diagnosed with PD were included in the investigation. Subjects of NC were included who had no history of neurological disease, no symptom of PD and no disorder of cognitive function and movement function. Diagnosis of PD was according to the clinical criteria of Movement Disorder Society (MDS; Postuma et al., 2015). PD patients included: those aged over 30 years old, no less than 1 year of disease duration, had received a stable dose of levodopa medication treatment for at least 30 days, without cardiovascular disease and respiratory disease, nor with a history of surgical operations or embedded with a pacemaker in the body. PD patients with severe symptoms of dementia, anxiety and depression were excluded. All of the PD patients were in a medication-on state during experimental data collection and no drug-naïve patient was included in this study. Both NC and PD subjects were recruited by Guangzhou First People’s Hospital from May 2017 to September 2018. This study was approved by the Institutional Review Board (IRB) of Guangzhou First People’s Hospital. Informed written consents were obtained from all subjects.

### Clinical Assessments

Clinical assessments, including motor and non-motor symptoms, were measured across all subjects with PD. Hoehn & Yahr (H&Y) scale (Hoehn and Yahr, 1998) was collected from subjects of PD group to evaluate the severity of PD symptoms, with classifications of stages 1–5, with a higher H&Y stage indicating an advanced state of PD. Additionally, cognitive function and motor function were also measured. For both NC and PD, cognitive-related measurement was identified by the Frontal Assessment Battery (FAB) containing six sub-items that are associated with the frontal cortex (Dubois et al., 2000). Movement-related assessment was evaluated from PD (medication-on) by the motor part (Part three) of Unified Parkinson’s Disease Rating Scale (UPDRS-III), which was developed by the MDS (Goetz et al., 2008). Higher UPDRS-III scores indicated decreased movement ability.

### Data Acquisition

Two groups of subjects (35 NC and 35 PD) participated in the MRI scanning by 3.0T SIEMENS MRI machine system. All of the subjects were required to lie quietly and stayed awake with eye closed during the whole process of scanning. All of the PD patients were in medication-on state when the fMRI was performed. Functional images and structural images of the brain were obtained. The resting-state functional images were obtained with the following parameters: repetition time (TR) of 2,000 ms, echo time (TE) of 21 ms, slice thickness of 4 mm, acquisition matrix of 64 × 64; flip angle (FA) of 78° and pixel spacing of 3.5 mm × 3.5 mm. Structural T1-weighted images were scanned with parameters: 1,900/2.22 ms TR/TE, acquisition matrix of 256 × 215, 9° FA, pixel spacing of 0.488 × 0.488 and 1 mm slice thickness.

### Data Preprocessing

Data preprocessing was implemented on MATLAB platform based on toolkit package of DPABI (Yan et al., 2016) and Statistical Parametric Mapping (SPM12^1^). Preprocessing procedures included: removal of the first 10 of 220 time points; slice timing adjustment (33 slices); realign; segmentation using new segment (Ashburner and Friston, 2005) and Diffeomorphic Anatomical Registration through Exponentiated Lie Algebra (DARTEL; Ashburner, 2007); regression of nuisance covariates including while matter, cerebrospinal fluid and Friston 24 parameters of head motions (Friston et al., 1996; Satterthwaite et al., 2013; Yan et al., 2013); spatial normalization with resampling of 3 mm × 3 mm × 3 mm to Montreal Neurological Institute (MNI) space by DARTEL (Ashburner, 2007); temporal filtering with low frequency band pass of 0.01–0.1 Hz and linear detrend removal. Subjects with maximal translations exceeded 2.5 mm or rotations over 2.5° were excluded from analysis. According to this exclusion criteria, no subjects were excluded. Additionally, the mean framewise displacement (FD) Jenkinson (Jenkinson et al., 2002) was calculated, representing the head motions of every subject. No significant difference of FD Jenkinson between two groups was observed by two-sample *t*-test (*p* = 0.1294).

### Global Signal Synchronization—Degree Centrality

DC is a voxel-wise measurement calculating the functional connectivity density between a voxel with the other voxels within the mask (Buckner et al., 2009). Pearson correlation is employed to evaluate the connectivity strength of all pairs of voxels. DC is conventionally calculated as weighted-sum DC or binarized-sum DC. The weighted-sum DC is defined as summing up the correlation coefficients that reach a given threshold, whereas the binarized-sum DC is defined as summing up the number of correlation coefficient that reaches a given threshold. Therefore, DC is also named global functional connectivity density, long range functional connectivity and global signal synchronization (Tomasi and Volkow, 2012). In this study, based on the preprocessed functional image, binarized-sum DC was calculated and the threshold was set at 0.3. For standardization, the DC maps of all subjects were transformed into Z-maps by subtracting the global mean value and then divided by standard deviation. After standardization transformation, the Z-maps were then smoothed with 4 mm of full width at half maximum (FWHM). The smoothed Z-maps were applied to the subsequent statistical analysis and correlative analysis.

### Statistical and Correlative Analysis

To explore global signal synchronization differences between NC and PD, a two-sample *t*-test was performed on DC maps of two groups, with age, sex, education time and mean FD Jenkinson as covariates within the mask of gray matter. The resultant statistical T-map was corrected with multiple comparisons of Gaussian Random Field (GRF) within gray matter mask, with voxel *p* < 0.005 and cluster *p* < 0.05, two-tailed test (*T* > 2.91, cluster size > 1,350 mm^3^).

The brain regions showing significant group differences were extracted as regions of interest (ROIs) to explore the neural correlates between global signal synchronization (DC) and cognitive function (FAB) as well as movement function (UPDRS-III). DC signals were extracted from ROIs by averaging the signals of all voxels within ROI. Pearson correlation (statistical significance level *p* < 0.05) was applied to calculate the correlations between DC and cognitive/movement function. Moreover, the correlation analysis was also analyzed across the whole brain, within Automated Anatomical Labeling (AAL) template, which contains 116 brain regions, including 90 cerebrum regions and 26 cerebellum regions (Tzourio-Mazoyer et al., 2002).

## Results

### Demographic Characteristics and Clinical Assessments

Statistical results of demographic characteristics and clinical measurements were summarized in Table 1. No group difference (*p* > 0.05) was observed on age or mean FD Jenkinson. Significant group differences were demonstrated in FAB scores (*p* < 0.0001) and education time (*p* = 0.0277). NC were shown to have higher FAB scores and longer education time than PD (Table 1). It should be noted that the FAB scores were obtained from 31 out of 35 PD patients because four PD patients refused to do the FAB assessment.

**Table 1:** Demographic characteristics and clinical assessments.

	NC (*n* = 35)	PD (*n* = 35)	Statistical *p*-value
Age (years)	60 ± 6	63 ± 12	0.0989
Sex (female/male)	24/11	18/17	NA
Education time	11.08 ± 2.84	9.43 ± 3.31	0.0277
(years)
Hand dominance	0/35	2/33	NA
(left/right)
Disease duration	NA	4.19 ± 3.97	NA
(years)
H&Y scores	NA	2.44 ± 0.72	NA
UPDRS-III scores	NA	31.93 ± 14.56	NA
(medication-on)
FAB scores	17.17 ± 1.34	15.16 ± 2.44 (*n* = 31)	<0.0001
Levodopa equivalent	NA	431.95 ± 383.33	NA
daily dose (mg)
Mean FD (mm)	0.088 ± 0.064	0.069 ± 0.031	0.1284

*NC, normal controls; PD, Parkinson’s disease; FD, framewise displacement of Jenkinson; H&Y, Hoehn & Yahr; UPDRS-III, Unified Parkinson’s Disease Rating Scale (part three); FAB, Frontal assessment battery; NA, not applicable. Data was noted as average ± standard deviation. Statistical *p*-value was obtained by two-sample *t*-test with significance level of *p* < 0.05*.

### Differences of Global Synchronizations

**Figure 1:**
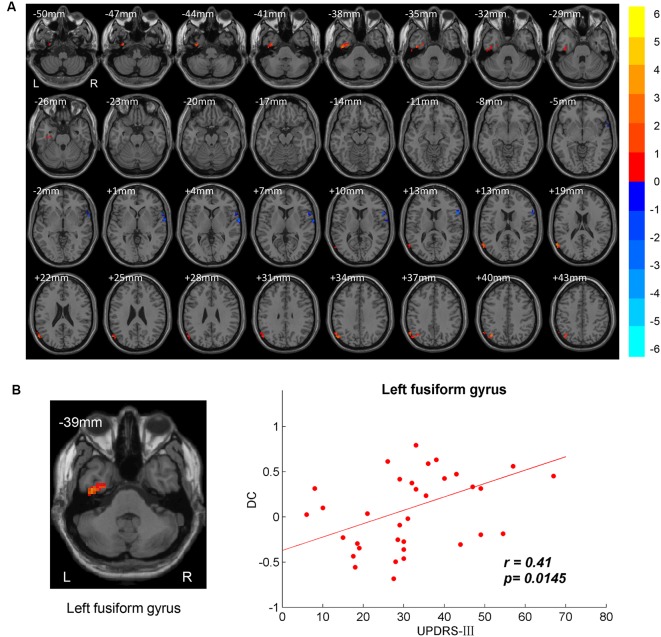
Between-group degree centrality (DC) differences. **(A)** T-maps of DC differences between normal controls (NC) and patients with Parkinson’s disease (PD). Multiple comparison corrections were implemented by Gaussian Random Field (GRF) with voxel *p* < 0.005 and cluster *p* < 0.05 within gray matter mask (*T* > 2.91, cluster size > 1,350 mm^3^). The color bar indicated the statistical *t*-value. Warm/Cool overlays indicated increased/decreased DC in PD. L/R = left/right hemisphere. **(B)** Positive correlation between DC and motor part of Unified Parkinson’s Disease Rating Scale (UPDRS-III) in left fusiform gyrus.

DC differences between NC and PD were implemented by two-sample *t*-test. The significant brain regions were showed in Table 2, Figure 1A. The survival voxels of brain regions were identified based on CUI Xu’s XjView^2^. For the PD group, increased DC was observed in left fusiform gyrus extending to inferior temporal gurus (ITG), left middle temporal gyrus (MTG) and angular gyrus, whereas it was decreased in right interior opercular-frontal gyrus (IFGoper) extending to superior temporal gyrus (STG).

**Table 2:** Brain regions showing significant degree centrality (DC) differences between normal controls (NC) and patients with Parkinson’s disease (PD).

Side	Brain regions	Brodmann area	Cluster size (mm^3^)	Peak MNI coordinates (*x y z*)	Peak *t*-value
Left	Fusiform gyrus/Inferior temporal gyrus	20	1,890	−42 −15 −39	4.85
Left	Middle temporal gyrus/Angular gyrus	39	2,754	−60 −63 18	4.73
Right	Inferior opercular-frontal gyrus/Superior temporal gyrus	44/48	1,620	57 15 12	−4.59

*MNI, Montreal Neurological Institute. Positive/Negative statistical *t*-value indicated increased/decreased DC in PD*.

### Correlative Analysis

**Figure 2:**
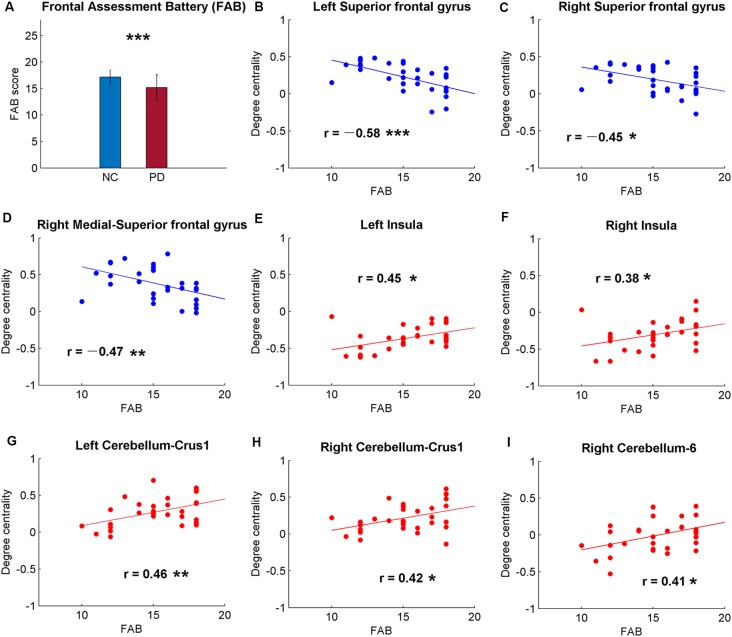
Significant correlations between cognitive function and the global signal synchronizations within the whole brain [Automated Anatomical Labeling (AAL) templates] in patients with PD. **(A)** The Frontal Assessment Battery (FAB) scores of NC and PD. Significant correlations between FAB and DC in bilateral superior frontal gyrus (SFG; **B,C**), right medial-SFG **(D)**, bilateral insula **(E,F)**, bilateral cerebellum-curs1 **(G,H)** and right cerebellum-6 **(I)**. Significance notations: *p < 0.05, **p < 0.01, ***p < 0.005. The dots and lines were demonstrated in red/blue color for positive/negative correlations.

**Figure 3:**
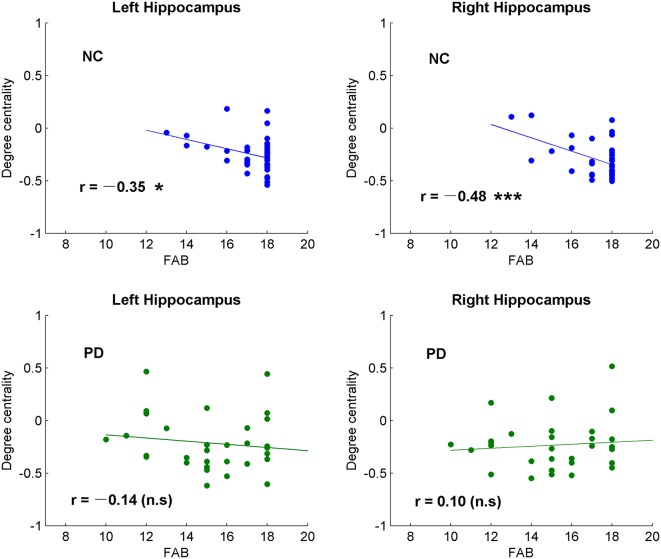
Correlative analysis between cognitive function and the global signal synchronizations in bilateral hippocampus in both NC and patients with PD. Significance notations: *p < 0.05, ***p < 0.005, n.s = no significance. The dots and lines were demonstrated in blue/green color for negative/no correlations.

**Figure 4:**
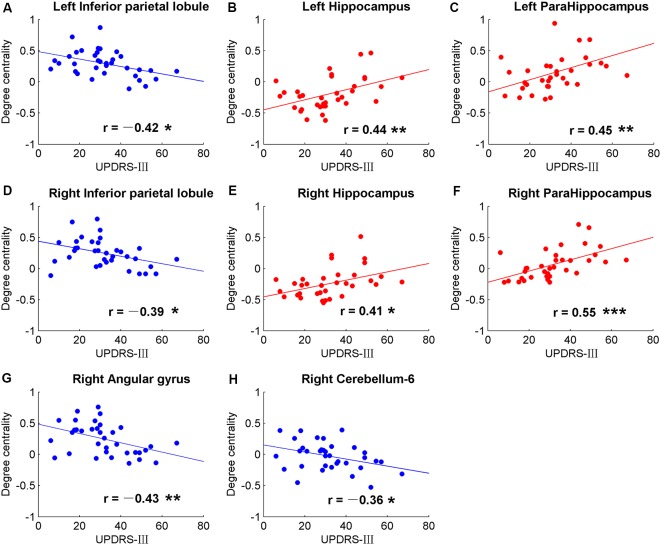
Significant correlations between movement measurements and the global signal synchronizations within the whole brain (AAL templates) in patients with PD. Correlations between Part three of UPDRS-III and DC in bilateral inferior parietal lobule (IPL; **A,D**), right angular gyrus **(G)**, right cerebellum-6 **(H)**, bilateral hippocampus **(B,E)** and bilateral para-hippocampus gyrus **(C,F)**. The dots and lines were demonstrated in red/blue color for positive/negative correlations.

The brain regions showing significant DC differences (Table 2) between NC and PD were extracted as ROIs. The correlative analysis between DC and cognitive/movement functions was performed within these brain regions. Positive correlation was observed between DC and UPDRS-III in left fusiform gyrus (*r* = 0.41, *p* = 0.0145; Figure 1B), while no correlation (*p* > 0.05) was observed between DC and FAB scores. Additionally, the neural correlations of cognitive function (FAB) and movement function (UPDRS-III) were examined across the whole brain within AAL templates. The significant results were demonstrated in Figures 2–4.

Among the correlative analysis between DC and FAB scores in PD, negative correlations were observed in bilateral superior frontal gyrus (SFG; Figures 2B,C) and right medial SFG (Figure 2D). The results were shown to be positive in bilateral insula (Figures 2E,F), bilateral cerebellum-crus1 (Figures 2G,H) and right cerebellum-6 (Figure 2I). The results were insignificant (*p* > 0.05) in NC within these regions. Conversely, significant negative results (*p* < 0.05) were observed in the bilateral hippocampus in NC, while the results were unobvious (*p* > 0.05) in PD (Figure 3).

Significant correlative analysis between DC and movement function (UPDRS-III) of PD within AAL templates are demonstrated in Figure 4. Negative correlations were observed in bilateral inferior parietal lobule (IPL; Figures 4A,D), right AG (Figure 4G), and right cerebellum-6 (Figure 4H). DC were positively correlated with UPDRS-III in the bilateral hippocampus (Figures 4B,E) and para-hippocampus gyrus (Figures 4C,F).

## Discussion

In this study, the indicator of DC was adopted to compare the difference of global signal synchronizations of spontaneous brain activity between PD and NC. In addition, the neural correlations between global synchronizations and cognitive/movement functions were explored across the whole brain within AAL templates. Significant results were observed in both analysis of group DC differences and neural correlations.

### More Sticky to Default Mode State in Parkinson’s Disease

Significant DC differences between PD and DC were observed in left fusiform gyrus extending to ITG, left MTG/angular gyrus and right IFGoper/STG (Table 2, Figure 1A). Posterior MTG and angular gyrus, together with medial prefrontal cortex (MPFC) and precuneus/posterior cingulate cortex (PCu/PCC), are identified as critical brain regions that constitute default mode network (DMN; Raichle et al., 2001; Laird et al., 2009). Brain activities in DMN are task-negative, which means deactivations during task-related state and activations in resting state (Raichle et al., 2001). DMN is also thought to be associated with self-referential processing (de Groot et al., 2000; Gusnard and Raichle, 2001). In our results, increased DC was demonstrated in left MTG and angular gyrus compared to in the NC group in a resting state. Dysfunctional DMN was also reported in other neuropsychiatric disorders like schizophrenia (Calhoun et al., 2008) and Alzheimer’s disease (Lustig et al., 2003). Additionally, less deactivations of DMN in PD were also observed in executive tasks than those in NC (Van Eimeren et al., 2009). DC evaluates global synchronizations and global functional connectivity density, therefore, higher DC activities indicate higher binding between inter-regions collaboration. Therefore, higher global synchronizations of DMN in PD may result in decreased ability to be self-referential, more likely to remain the default mode state and less control of interactions between brain regions.

Cognitive impairment is commonly present in PD patients. A VBM study on PD reports gray matter atrophy in left fusiform gyrus, and the fusiform atrophy is associated with poor memory (Camicioli et al., 2009). Fusiform gyrus is famous for its face area and face perception (Kanwisher et al., 1997; George et al., 1999). PD patients have an impairment in recognizing facial expression and visuospatial dysfunctions (Levin et al., 1991; Sprengelmeyer et al., 2003). In our results, increased DC was also observed in left fusiform gyrus and the DC in the fusiform is positively correlated with movement function for PD patients (Figure 1, Table 2). Consistent with previous study, increased activity is shown in the fusiform gyrus in response to the paradigm of facial perception (Cardoso et al., 2010). Visuospatial dysfunctions have an impact on the movement of PD especially those with freezing gait. The correlation between DC in fusiform gyrus and movement function may be explained by impaired vision in balance (Day and Guerraz, 2007).

### Neural Correlations of Cognitive and Movement Function in Parkinson’s Disease

Patients with PD are not only reported with abnormalities of motor symptoms but also with decreased cognitive function (Dubois and Pillon, 1996; Kehagia et al., 2010). As expected, the FAB scores of PD were significantly lower than NC (*p* < 0.0001; Figure 2A), suggesting decreased frontal-related of cognitive function in PD. The correlative analysis between global synchronizations and cognitive function scores across the whole brain showed significant correlations in frontal cortex, insula and cerebellum in PD patients (Figure 2), while there was no significant result in NC. The DC of PD was negatively correlated with FAB scores in bilateral SFG and medial SFG, and positively correlated with bilateral insula and cerebellum. The frontal cortex is always associated with cognitive and executive functions (Miller and Cohen, 2001). Cortical thickness changes and gray matter volume reductions were reported in frontal cortex of PD (Pan et al., 2012; Tessitore et al., 2016). Therefore, negative correlations between DC and FAB scores reveal altered frontal-cognitive function in PD.

Contrary to frontal cortex, the correlations between DC and FAB scores were positive in the insula (Figure 2). Though the insula is a well-known brain region that is associated with self-representation (Burgmer et al., 2013) and awareness (Craig, 2009), it is also suggested to be related to non-motor symptoms of PD (Christopher et al., 2014a). In addition, patients with PD were observed with reduced gray matter volume in insula (Pan et al., 2012) and decreased DC in bilateral insula in medication-off state (Zhong et al., 2018). Insular dysfunction is also related to PD with cognitive impairment (Christopher et al., 2014b). These findings suggest higher DC in insula of PD suggests higher cognitive function.

Positive correlations between DC and FAB scores were also shown in the cerebellum (Figure 2). However, negative correlations were observed between DC and movement function (UPDRS-III) in PD (Figure 4). Cerebellum is both associated with sensorimotor processing (Baumann et al., 2015; Kansal et al., 2017) and cognitive/emotional processing (Schmahmann, 2010; Adamaszek et al., 2017). Decreased DC in cerebellum-6 was also observed in PD (Wang et al., 2018). Positive results in neural correlations of cognitive function and negative neural correlations of movement function were found in the cerebellum, suggesting that the cerebellum plays both cognitive and motor function roles in the pathology of PD.

The hippocampus is well known for its role in memory (Squire, 1992). Interestingly, negative correlations were observed between DC and FAB scores in the hippocampus of NC, while no correlation was observed (*p* > 0.05) in PD (Figure 3). Moreover, positive correlations were shown between DC and movement function (UPDRS-III) in the hippocampus/para-hippocampus in PD (Figure 4). These findings seem to suggest that the global synchronizations in the hippocampus reveal both cognitive and movement functions of PD. Hippocampus atrophy is found in PD patients with depressive symptoms, cognitive impairment and dementia (van Mierlo et al., 2015; Delgado-Alvarado et al., 2016). A longitudinal study on PD patients suggests that the hippocampus is related to the progression of cognitive impairment and dementia (Kandiah et al., 2014). Decoupled correlation between hippocampal DC and FAB scores of PD (Figure 3) may imply cognitive decline in PD. The findings of PD studies in reference to hippocampus/para-hippocampus are extensively focused on non-motor symptoms, mostly on depression, cognitive decline and memory impairment, and are rarely related to motor symptoms or movement function. The hippocampus is involved in motor tasks and perturbed movements (Devan et al., 2015; Kerr et al., 2017) which are not general movements. No direct evidence supports the influence of hippocampus on motor effect in PD. Our finding of increased hippocampal signal synchronization comes with increased motor performance in PD (Figure 4) suggests that the hippocampus has an important role in the motor symptoms of PD.

In our results, negative correlations between DC and movement function (UPDRS-III) were observed in bilateral IPL (Figure 4). Altered brain activities of parietal lobe are widely reported in PD studies. Decreased inter-hemispheric functional connectivity in IPL was demonstrated in PD and negative neural correlation was observed in interaction with motor scores (Li et al., 2018). Meta-analysis also suggests that IPL is a robust brain region that showed significant differences in regional synchronizations between PD and NC (Pan et al., 2017a). Therefore, IPL may be a critical brain region in the motor symptom of PD.

The findings of this study suggest the global synchronizations of the fusiform, hippocampus and cerebellum-6 are critical brain regions for both cognitive function and movement function of PD. However, the exact pathology remains unclear and needs further studies on it.

## Conclusion

Alterations of global synchronization in the left fusiform gyrus and right opercular-frontal cortex reveal altered cognitive and movement functions in PD. The findings of neural correlations suggest that the global functional connectivity in fusiform gyrus, cerebellum and hippocampus are critical in the identification of cognitive and movement functions in PD. This study provides new insights on the interaction among global coordination of brain activity, cognitive function and movement function in PD.

## Ethics Statement

This study was approved by the Institutional Review Board (IRB) of Guangzhou First People’s Hospital. Informed written consents were obtained from all subjects.

## Author Contributions

ML and YL wrote the manuscript and designed the experiment. YL, XW and YX conceived the idea and performed the literature review. YL, ML, HC, GH and SY performed the data analysis. HC, GH, XR, ZL and XW contributed to data collection. All authors reviewed the manuscript and joined the discussion of the manuscript.

## Conflict of Interest Statement

ZL was employed by the company GYENNO Technologies Co., Ltd.

## Footnotes

^1^http://www.fil.ion.ucl.ac.uk/spm/

^2^http://www.alivelearn.net/xjview/
